# H1N1 infection and thyroid storm

**DOI:** 10.4103/1817-1737.69119

**Published:** 2010

**Authors:** Viroj Wiwanitkit

**Affiliations:** *Wiwanitkit House, Bangkhae, Bangkok – 10160, Thailand. E-mail: wviroj@yahoo.com*

Sir,

I read the recent publication on H1N1 infection and thyroid storm with great interest.[1] Baharoon reported a case of H1N1 infection inducing thyroid storm and noted that it was the first case report. Indeed, it is no doubt that thyroid storm can be induced by infection. There are some points to be noted on this work. This case might not be the first case report as mentioned by the author. A similar case was already published by Oguz *et al*.[2] A conclusion that H1N1 infection directly induces thyroid storm should be carefully considered since the pathophysiologic process is is yet to be clarified. Only a potential impact of H1N1 influenza infection in thyrotoxic patients might be concluded.[2]
